# Suppression of Parkinsonian Beta Oscillations by Deep Brain Stimulation: Determination of Effective Protocols

**DOI:** 10.3389/fncom.2018.00098

**Published:** 2018-12-11

**Authors:** Eli J. Müller, Peter A. Robinson

**Affiliations:** ^1^School of Physics, The University of Sydney, Sydney, NSW, Australia; ^2^Center for Integrative Brain Function, The University of Sydney, Sydney, NSW, Australia

**Keywords:** deep brain stimulation, neural field theory, Parkinson's disease, cortex, thalamus, basal ganglia

## Abstract

A neural field model of the corticothalamic-basal ganglia system is developed that describes enhanced beta activity within subthalamic and pallidal circuits in Parkinson's disease (PD) via system resonances. A model of deep brain stimulation (DBS) of typical clinical targets, the subthalamic nucleus (STN) and globus pallidus internus (GPi), is added and studied for several distinct stimulation protocols that are used for treatment of the motor symptoms of PD and that reduce pathological beta band activity (13–30 Hz) in the corticothalamic-basal ganglia network. The resulting impact of DBS on enhanced beta activity in the STN and GPi, as well as cortico-subthalamic and cortico-pallidal coherence, are studied. Both STN-DBS and GPi-DBS are found to be effective for suppressing peak STN and GPi power in the beta band, with GPi-DBS being slightly more effective in both the STN and the GPi for all stimulus protocols tested. The largest decrease in cortico-STN coherence is observed during STN-DBS, whereas GPi-DBS is most effective for reducing cortico-GPi coherence. A reduction of the pathologically large STN connection strengths that define the parkinsonian state results in enhanced 6 Hz activity and could thus represent a compensatory mechanism that has the side effect of driving parkinsonian tremor-like oscillations. This model provides a method for systematically testing effective DBS protocols that agrees with experimental and clinical findings. Furthermore, the model suggests GPi-DBS and STN-DBS have distinct impacts on elevated synchronization between the basal ganglia and motor cortex in PD.

## 1. Introduction

Deep brain stimulation (DBS) has become an effective treatment for Parkinson's disease (PD), dystonia, and tremor (Benabid et al., 1996; Krack et al., 1998; Vidailhet et al., 2005; Moro et al., 2010), and its use is expanding to other neurological and neuropsychiatric conditions (Boon et al., 2007; Figee et al., 2013). In DBS treatments for Parkinson's disease, high frequency (>80 Hz) electrical stimulation is applied as a series of pulses via a chronically implanted electrode, typically in either the globus pallidus internus (GPi), subthalamic nucleus (STN) (Rodriguez-Oroz et al., 2005), or the ventral intermediate nucleus of the thalamus (Cury et al., 2017). Despite the efficacy of these treatments, the underlying therapeutic mechanisms of DBS remain poorly understood. Furthermore, it is unclear what stimulation protocols and target regions are most effective. A framework for systematically determining efficacious stimulus parameters would prove valuable since these are hard to optimize for an individual and have been estimated by trial and error to date.

Pathologically synchronous activity within the corticothalamic-basal ganglia network is a prominent feature of Parkinson's disease and its animal models (Brown et al., 2001; Tachibana et al., 2011). Enhanced 4−8 Hz and 13−30 Hz coherent oscillations are observed within and between the basal ganglia (BG), thalamus and motor cortex (Marsden et al., 2001; Levy et al., 2002a,b; Williams et al., 2002; Timmermann et al., 2003; Kühn et al., 2005; Wang et al., 2005; Rivlin-Etzion et al., 2006; Weinberger et al., 2006; Tass et al., 2010). STN activity in the 13−30 Hz beta range has also been shown to correlate with the motor symptoms of human PD (Neumann et al., 2016; Beudel et al., 2017). Furthermore, studies of PD patients combining either simultaneous EEG (electroencephalography), ECoG (electrocorticography), or magnetoencephalography (MEG) and intracranial LFP (local field potential) recordings have shown that cortico-STN coherence (Fogelson et al., 2005b; Litvak et al., 2010; Hirschmann et al., 2011) and cortico-GPi coherence (Wang et al., 2018) within the beta band may be of physiological and pathological significance.

Therapeutically effective dopamine supplementation and deep brain stimulation in PD patients reduce both the power of beta activity in the STN (Beudel et al., 2017) and GPi (Wang et al., 2018), as well as cortico-STN coherence (Marsden et al., 2001; Williams et al., 2002; Kühn et al., 2008; Hirschmann et al., 2013; Oswal et al., 2016) and cortico-GPi coherence (Wang et al., 2018) about these frequencies.

A biophysical theory was previously developed that incorporated both a description of both PD as well as the impact of deep brain stimulation on parkinsonian states (Müller and Robinson, 2018). Here we further develop this theory and compare the effectiveness of distinct stimulation protocols and target regions for suppressing pathologically coherent beta activity in the corticothalamic-basal ganglia network.

## 2. Materials and Methods

A description of the CTBG model and how DBS is incorporated is given next.

### 2.1. Corticothalamic-Basal Ganglia Model

Physiologically based neural field theory enables a tractable framework for the analysis of large-scale neuronal dynamics by averaging microscopic structure and activity (Wilson and Cowan, 1973; Nunez, 1974; Jirsa and Haken, 1996; Wright and Liley, 1996; Robinson et al., 1997). Neural field theory incorporates realistic anatomy of neural populations, nonlinear neural response, interpopulation connections; and dendritic, synaptic, cell-body, and axonal dynamics (Wilson and Cowan, 1973; Wright and Liley, 1996; Robinson et al., 1997, 1998, 2001, 2002, 2005; Rennie et al., 1999; Deco et al., 2008). Neural field models have been successful in accounting for many characteristic states of brain activity, including sleep stages, eyes-open and eyes-closed waking, nonlinear seizure dynamics, anesthesia, and many other phenomena (Jirsa and Haken, 1996; Robinson et al., 1997, 1998, 2002; Steyn-Ross et al., 2004; Liley and Bojak, 2005; Breakspear et al., 2006; Roberts and Robinson, 2008).

**Figure 1:**
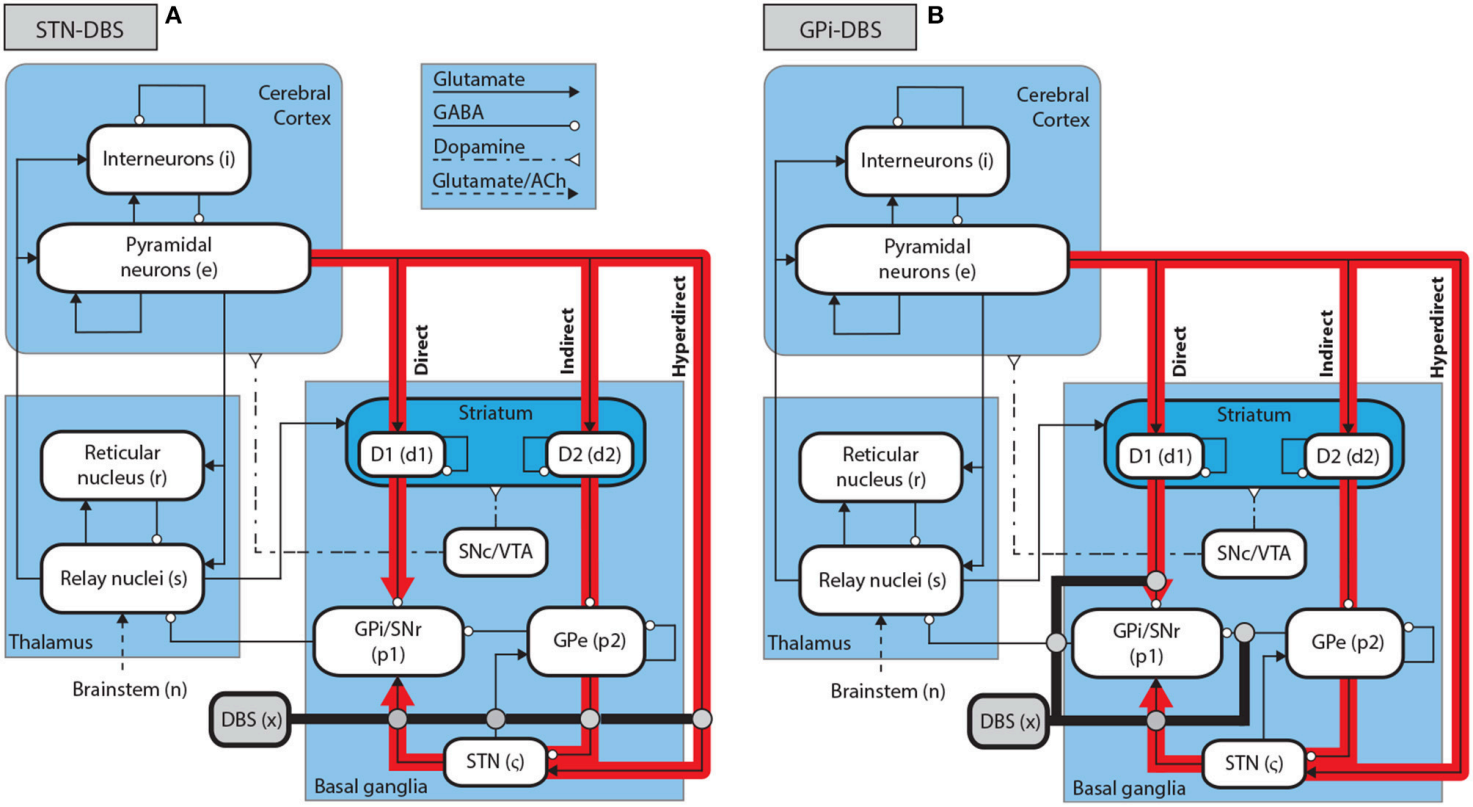
Schematic of stimulation targets and the corticothalamic-basal ganglia system. **(A)** Schematic of STN-DBS in the CTBG system. **(B)** Schematic of GPi-DBS in the CTBG system.

The corticothalamic-basal ganglia system is divided into nine distinct populations over three brain regions, as shown in Figure 1. The cerebral cortex contains populations of excitatory pyramidal neurons, *e*, and inhibitory interneurons, *i*. The thalamus is divided into an excitatory population for the specific relay nuclei (SRN), *s*, and an inhibitory population for the thalamic reticular nucleus (TRN), *r*. The basal ganglia (BG) contain two inhibitory populations within the striatum, one expressing the D1 dopamine receptor, *d*_1_, and one expressing the D2 dopamine receptor, *d*_2_. The striatum projects to two inhibitory populations, the globus pallidus pars externa, *p*_2_, and a population representing the globus pallidus pars interna and substantia nigra pars reticulata, *p*_1_. The subthalamic nucleus (STN) is represented by an excitatory population, ζ. Deep brain stimulation is represented as an external input source, *x*. The substantia nigra pars compacta (SNc) and ventral tegmental area (VTA) are not explicitly included as populations within the present model, since they are not the focus of this study, but are shown in Figure 1 as an indication of the neural pathways affected by dopamine.

### 2.2. Firing Rates

The mean firing rate, *Q*_*a*_(**r**, *t*) of a neural population at position **r** and time *t* can be approximately related to its mean membrane potential, *V*_*a*_(**r**, *t*), by Wilson and Cowan (1972) and Freeman (1975)

(1) $$\begin{array}{rc} Q_{a}\left(\text{r},t\right) & =S_{a}\left[V_{a}\left(\text{r},t\right)\right], \end{array}$$

(2) $$\begin{array}{r} =\frac{Q_{a}^{\text{max}}}{1+\text{exp}\left[-\left\{V_{a}\left(\text{r},t\right)-\theta_{a}\right\}/\sigma^{\prime }\right]}, \end{array}$$

where $Q_{a}^{\text{max}}$ is the maximal firing rate, *V*_*a*_ is the average membrane potential relative to resting, θ_*a*_ is the mean neural firing threshold, and $\sigma^{\prime }\pi /\sqrt{3}$ is the standard deviation of this threshold.

### 2.3. Axonal Propagation

A number of experimental studies have revealed waves of neural activity spreading across the cortex (Burns, 1951; Nunez, 1974; Chervin et al., 1988; Golomb and Amitai, 1997), which have been analyzed theoretically (Beurle, 1956; Nunez, 1995; Jirsa and Haken, 1996, 1997; Robinson et al., 1997; Bressloff, 2001; Bressloff et al., 2003; Deco et al., 2008). This propagating activity is represented as a field of mean spike rates in axons, ϕ_*a*_, where ϕ_*a*_ is approximately related to *Q*_*a*_ by the damped wave equation

(3) $$\begin{array}{r} D_{a}\left(\text{r},t\right)\phi_{a}\left(\text{r},t\right)=Q_{a}\left(\text{r},t\right), \end{array}$$

where

(4) $$\begin{array}{r} D_{a}\left(\text{r},t\right)=\frac{1}{\gamma_{a}^{2}}\frac{\partial^{2}}{\partial t^{2}}+\frac{2}{\gamma_{a}}\frac{\partial }{\partial t}+1-r_{a}^{2}\nabla^{2}. \end{array}$$

Here γ_*a*_ = *v*_*a*_/*r*_*a*_ represents the damping rate, where *v*_*a*_ is the propagation velocity in axons and *r*_*a*_ is the characteristic axonal length for the population. The propagation of these waves is primarily via the relatively long-range white matter axons of excitatory cortical pyramidal neurons. Later in our model the local interaction approximation *r*_*b*_≈0 is made for *b* = *i, r, s, d*_1_, *d*_2_, *p*_1_, *p*_2_, ζ due to the short ranges of the corresponding axons which implies ϕ_*b*_(**r**, *t*) = *Q*_*b*_(**r**, *t*) for these populations (Robinson et al., 1997, 2001, 2002, 2004; Rennie et al., 1999; Rowe et al., 2004).

### 2.4. Synaptodendritic and Somatic Response

The mean soma potential *V*_*a*_ of a population *a* at position **r** and time *t* is given by sum of the postsynaptic potentials (PSPs):

(5) $$\begin{array}{r} V_{a}\left(\text{r},t\right)=\underset{b}{\sum }V_{ab}\left(\text{r},t\right), \end{array}$$

where *V*_*ab*_(**r**, *t*) is the postsynaptic potential generated by projections arriving at population *a* from population *b*. The influence of incoming spikes to population *a* from population *b* is weighted by a connection strength parameter, ν_*ab*_ = *N*_*ab*_*s*_*ab*_, where *N*_*ab*_ is the mean number of connections between the two populations and *s*_*ab*_ is the mean strength of response in neuron *a* to a single spike from neuron *b*. The postsynaptic potential response in the dendrite is given by

(6) $$\begin{array}{r} D_{\alpha \beta }V_{ab}\left(\text{r},t\right)=\nu_{ab}\left(\text{r},t\right)\phi_{ab}\left(\text{r},t-\tau_{ab}\right), \end{array}$$

where τ_*ab*_ is the average axonal delay for the transmission of signals to population *a* from population *b*. The operator *D*_αβ_ describes the time evolution of *V*_*ab*_ in response to synaptic input, and is given by

(7) $$\begin{array}{r} D_{\alpha \beta }=\frac{1}{\alpha \beta }\frac{d^{2}}{dt^{2}}+\left(\frac{1}{\alpha }+\frac{1}{\beta }\right)\frac{d}{dt}+1, \end{array}$$

where β and α are the overall rise and decay response rates of the synaptodendritic and soma dynamics.

### 2.5. Steady States

It has been shown that nominal brain activity is well characterized by perturbations about a mean value (Rennie et al., 2002; Robinson et al., 2002, 2003). Hence, we first determine the time independent states of the CTBG system. Following the approach of previous neural field models, excitatory and inhibitory synapses in the cortex are assumed proportional to the numbers of neurons (Wright and Liley, 1996; Braitenberg and Schüz, 1998). This random connectivity approximation results in ν_*ee*_ = ν_*ie*_, ν_*ei*_ = ν_*ii*_, and ν_*es*_ = ν_*is*_, which implies *V*_*e*_ = *V*_*i*_ and *Q*_*e*_ = *Q*_*i*_. Inhibitory population variables can then be expressed in terms of excitatory quantities and are thus not neglected even though they do not appear explicitly below. The steady states are obtained by setting all time derivatives to zero in Equations (3), (4), and (6), and considering the simultaneous zeros of the five functions

(8) $$\begin{array}{rc} F\left(V_{e}\right) & =V_{e}-\left[\left(\nu_{ee}+\nu_{ei}\right)\phi_{e}+\nu_{es}\phi_{s}\right], \end{array}$$

(9) $$\begin{array}{rc} F\left(V_{s}\right) & =V_{s}-\left[\nu_{se}\phi_{e}+\nu_{sr}\phi_{r}+\nu_{sp_{1}}\phi_{p_{1}}+\nu_{sn}\phi_{n}\right], \end{array}$$

(10) $$\begin{array}{rc} F\left(V_{d_{1}}\right) & =V_{d_{1}}-\left[\nu_{d_{1}e}\phi_{e}+\nu_{d_{1}s}\phi_{s}+\nu_{d_{1}d_{1}}\phi_{d_{1}}\right], \end{array}$$

(11) $$\begin{array}{rc} F\left(V_{d_{2}}\right) & =V_{d_{2}}-\left[\nu_{d_{2}e}\phi_{e}+\nu_{d_{2}s}\phi_{s}+\nu_{d_{2}d_{2}}\phi_{d_{2}}\right], \end{array}$$

(12) $$\begin{array}{rc} F\left(V_{p_{2}}\right) & =V_{p_{2}}-\left[\nu_{p_{2}d_{2}}\phi_{d_{2}}+\nu_{p_{2}p_{2}}\phi_{p_{2}}+\nu_{p_{2}\zeta }\phi_{\zeta }\right]. \end{array}$$

The roots of Equations (8)–(12) are computed numerically using the MATLAB function fsolve(), which implements the Levenberg-Marquardt (Marquardt, 1963) and trust-region methods (Coleman and Li, 1996), with a tolerance of 10^−15^ V.

### 2.6. Resonances and Gains

A linearized form of the CTBG model can be used to derive the transfer function of the system (van Albada and Robinson, 2009; van Albada et al., 2009; Müller et al., 2017). This is a function of the internal gains of the system, which represent the additional activity generated in postsynaptic nuclei per additional unit input activity from presynaptic nuclei, and are (Robinson et al., 1998, 2002)

(13) $$\begin{array}{r} G_{ab}=\rho_{a}\nu_{ab}, \end{array}$$

where

(14) $$\begin{array}{r} \rho_{a}=\frac{dQ_{a}}{dV_{a}}{|}_{V_{a}^{\left(0\right)}}=\frac{\phi_{a}^{\left(0\right)}}{\sigma^{\prime }}\left[1-\frac{\phi_{a}^{\left(0\right)}}{Q_{a}^{\text{max}}}\right]. \end{array}$$

### 2.7. Numerical Simulations

In this work we formulate the CTBG model for the case of spatial uniformity because the main point of comparison between model outputs and experimental recordings are the temporal structure of local field potential measurements of the parkinsonian BG. These recordings result from aggregate neural activity and their spatial structure has not been well explored.

All numerical simulations of the CTBG neural field model are performed using the NFTsim code package detailed by Sanz-Leon et al. (2018). This package is used to solve Equations (1)–(7) numerically for the spatially uniform case where the ∇^2^ in (4) can be omitted. The solutions to these delay differential equations are found using a fourth-order Runge-Kutta method (Sanz-Leon et al., 2018) with a time step of 10^−4^ s.

Nominal brain states have been found to exist near stable fixed points (Robinson et al., 2002), so all simulations in this work are performed with the system initialized to the low firing steady state found in section 2.5 using the parameters given in Table 1, unless otherwise specified.

**Table 1:** Nominal parkinsonian parameters (Müller and Robinson, 2018).

**Quantity**	**Value**	**Unit**
*r*	80	mm
σ′	3.3	mV
$\phi_{n}^{\left(0\right)}$	1	s^−1^
γ_*e*_	116	s^−1^
α	50	s^−1^
β	200	s^−1^
τ_*re*_, τ_*se*_	45	ms
τ_*es*_	35	ms
$Q_{e}^{\text{max}}$, $Q_{r}^{\text{max}}$, $Q_{p_{2}}^{\text{max}}$	300	s^−1^
$Q_{d_{1}}^{\text{max}}$, $Q_{d_{2}}^{\text{max}}$	65	s^−1^
$Q_{p_{1}}^{\text{max}}$	250	s^−1^
$Q_{\zeta }^{\text{max}}$	500	s^−1^
θ_*e*_	14	mV
θ_*r*_, θ_*s*_	13	mV
θ_*d*_1__, θ_*d*_2__	19	mV
θ_*p*_1__, θ_ζ_	10	mV
θ_*p*_2__	9	mV
ν_*ee*_	1.2	mV s
ν_*ei*_	−1.5	mV s
ν_*es*_	1.1	mV s
ν_*re*_	0.1	mV s
ν_*rs*_	0.1	mV s
ν_*se*_	1.5	mV s
ν_*sr*_	−0.1	mV s
ν_*s*_*p*__1__	−0.2	mV s
ν_*sn*_	0.5	mV s
ν_*d*_1_*e*_	0.1	mV s
ν_*d*_1_*s*_	1	mV s
ν_*d*_1_*d*_1__	−0.02	mV s
ν_*d*_2_*e*_	0.1	mV s
ν_*d*_2_*s*_	0.1	mV s
ν_*d*_2_*d*_2__	−0.02	mV s
ν_*p*_1_*d*_1__	−0.2	mV s
ν_*p*_1_*p*_2__	−0.02	mV s
ν_*p*_1_ζ_	1	mV s
ν_*p*_2_*d*_2__	−0.8	mV s
ν_*p*_2_*p*_2__	−0.2	mV s
ν_*p*_2_ζ_	2.4	mV s
ν_ζ*e*_	1.29	mV s
ν_ζ_*p*__2__	−0.2	mV s

### 2.8. Power Spectrum and Coherence

Power spectrums of population activity are computed numerically using the fast Fourier transform, as implemented in MATLAB's fft() function (Frigo and Johnson, 1998), averaged over 8 epochs.

The coherence of activity between two populations *Q*_*a*_(*t*) and *Q*_*b*_(*t*) is given by

(15) $$\begin{array}{r} \gamma_{ab}^{2}=\frac{|S_{ab}\left(f\right){|}^{2}}{S_{aa}\left(f\right)S_{bb}\left(f\right)}, \end{array}$$

where *S*_*ab*_(*f*) is the cross spectral density of the two signals, and *S*_*aa*_(*f*) and *S*_*bb*_(*f*) are the power spectral density functions for *Q*_*a*_(*t*) and *Q*_*b*_(*t*). The coherence is averaged over 150 epochs with 50% overlap.

### 2.9. Stimulation Protocols

Several different stimulation functions are used in this work which capture stimulus protocols found in clinical and experimental DBS. Each function defines an external pulse rate, ϕ_*x*_(*t*) made up of rectangular pulses with a width $t_{\text{width}}=2^{-11}$ s (~500 μs) and an amplitude $\phi_{x}^{\text{max}}=1$ s^−1^. The time-integral of ϕ_*x*_(*t*) over the pulse width *t*_width_ is the average number of additional spikes generated in the target axon by the applied stimulation. The external stimulus ϕ_*x*_(*t*) is then coupled to a target population *a* via a connection parameter ν_*ax*_.

**Figure 2:**
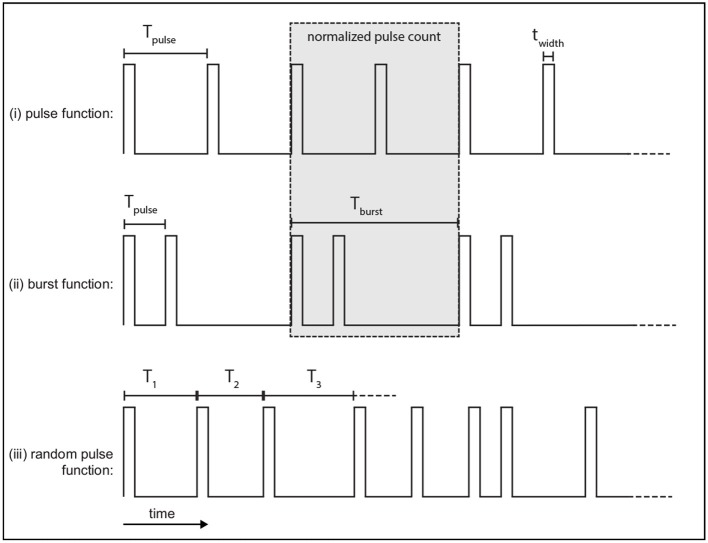
Example of stimulus protocols used. Each function consists of rectangular pulses which have a width *t*_width_. **(i)** The pulse function is a regular train of pulses with a frequency *f*_pulse_. **(ii)** The burst function is a train of pulses at frequency *f*_pulse_ that occur during each burst which is then followed by a quiescent interval before the next burst repeats at the burst frequency, *f*_burst_. **(iii)** The random pulse function is a train of pulses where each consecutive pulse interval is drawn from a random normal distribution parameterized by a mean period, *T*_pulse_, and standard deviation σ.

The pulse function, defined in Figure 2i, is a regular train of pulses that occur at a frequency *f*_pulse_ = 1/*T*_pulse_, which defines *T*_pulse_.

The burst function, defined in Figure 2ii, is a regular train of pulses and is parameterized by two distinct frequencies. Pulses occur during a bursting phase at a rate *f*_pulse_ = 1/*T*_pulse_, which is then followed by a quiescent interval before another burst occurs, with a burst frequency *f*_burst_ = 1/*T*_burst_.

In order to compare the pulse and burst functions, the number of pulses over the burst period, *T*_burst_, is normalized to the same value for both functions, as shown in the gray box of Figure 2. Here, this is done by setting a ratio of two pulses per burst with the burst frequency *f*_burst_ set equal to half the pulse function frequency. Each burst thus consists of two pulses which occur at *f*_pulse_ set to be twice the pulse function frequency. An example of this normalization is shown by the gray box in Figures 2i,ii. The total number of pulses over the burst period is two in this case.

In a later section the normalized pulse and burst functions are compared using two forms that we term low frequency (LF) and high frequency (HF) stimulation, as defined in Table 2.

**Table 2:** Stimulus frequency formats.

**Name**	**Parameters**
LF-pulse function	*f*_pulse_ = 128 Hz
LF-burst function	*f*_pulse_ = 256 Hz, *f*_burst_ = 64 Hz
HF-pulse function	*f*_pulse_ = 256 Hz
HF-burst function	*f*_pulse_ = 512 Hz, *f*_burst_ = 128 Hz

A variation of the pulse function is also used that introduces randomness by selecting consecutive inter-pulse intervals from a normal distribution parameterized by a mean interval and standard deviation. An example time series of this random function is given in Figure 2iii.

### 2.10. Stimulation Coupling

Following the approach of Müller and Robinson (2018), we assume that electric potentials applied via an implanted electrode result in activation of axons in close proximity to this electrode for typically used stimulus parameters. STN-DBS, as shown in Figure 1A, is then described by an external stimulus drive coupled to both the STN and also to the pallidal populations to which it projects. The strength of the stimulus to STN coupling is determined by adding together all intrinsic coupling parameters mapping pallidal and cortical inputs to the STN. The strengths of the stimulus couplings to the internal and external segments of the pallidus are set equal to the intrinsic STN-GPe and STN-GPi coupling strengths, respectively. The parameters used for STN-DBS coupling are explicitly defined in Table 3.

**Table 3:** Stimulus coupling parameters.

**Name**	**Parameter**	**Value**
STN-DBS:	ν_ζ*x*_ = ν_ζ*e*_+ν_ζ_*d*__2__	1.086 mV s
	ν_*p*_1_*x*_	1 mV s
	ν_*p*_2_*x*_	2.4 mV s
GPi-DBS:	ν_*p*_1_*x*_ = ν_*p*_1_*d*1_+ν_*p*_1_*p*_2__+ν_*p*_1_ζ_	0.78 mV s
	ν_*sx*_	−0.2 mV s

Similarly to STN-DBS, GPi-DBS is described by coupling stimulation to the GPi population, as shown in Figure 1B, and also to the SRN population in the thalamus which the GPi nuclei project to. The strength of the stimulus coupling to the GPi is determined by adding together all intrinsic coupling strengths mapping striatal, pallidal, and subthalamic inputs to the GPi. The strength of coupling the stimulus to the SRN is set equal to the intrinsic GPi-SRN coupling strength. The parameters used for GPi-DBS coupling are explicitly defined in Table 3.

## 3. Results

Here, the CTBG system is first configured to a parkinsonian state and related to experimental EEG and LFP spectral and coherence measures of PD. DBS of the STN and GPi is then incorporated to explore their respective impacts on this parkinsonian activity.

### 3.1. Beta Oscillations

**Figure 3:**
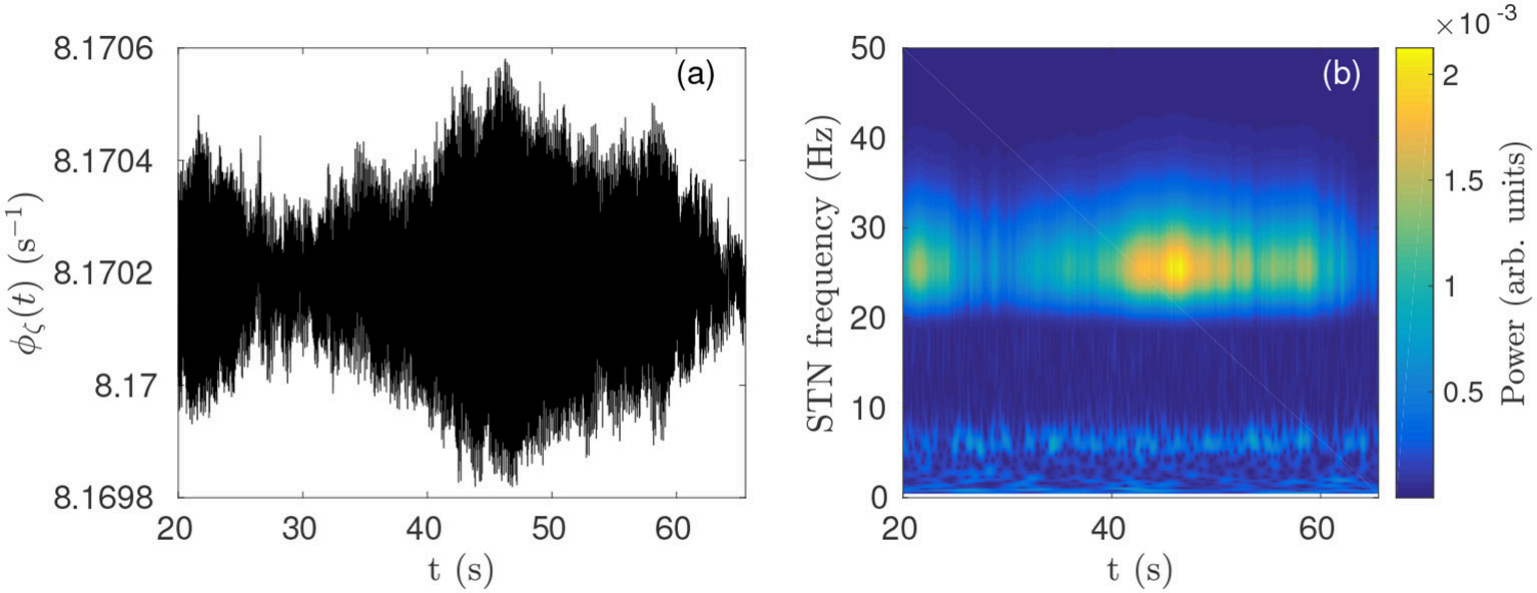
Parkinsonian STN beta activity where ν_ζ*e*_ = 1.3 mV s and ν_*sn*_ = 0.525 mV s and using Table 1 parameters. **(A)** Time series of STN firing rate. The high frequency oscillations appear as solid black in the time resolution plotted. **(B)** STN dynamic spectrum produced using a complex wavelet transform with a Morlet wavelet as implemented in MATLAB's (R2016b) cwtft() function (Torrence and Compo, 1998).

In previous work (Müller and Robinson, 2018) it was shown that a physiological model of the corticothalamic-basal ganglia system reproduces experimentally observed enhanced beta activity in parkinsonian states (Levy et al., 2000, 2002b) via pathologically strong gains within the STN-GPe and hyperdirect pathways, consistent with experimental inferences (Tachibana et al., 2011; Chiken and Nambu, 2013; Pavlides et al., 2015). Figures 3A,B demonstrate this enhanced beta band activity in the STN for parkinsonian parameters.

When the CTBG system is configured to a parkinsonian state using the parameters in Table 1, high cortico-STN and cortico-GPi coherence is observed about the beta resonance peak. Studies have shown that cortico-STN coherence (Fogelson et al., 2005b; Litvak et al., 2010; Hirschmann et al., 2011) and cortico-GPi coherence (Wang et al., 2018) within the beta band may play important roles in PD and could be relevant as biomarkers for motor symptoms.

A comparison of CTBG model power spectrums shows beta band power increases from the striatum to the thalamus, and from the thalamus to the STN. This trend in beta power is consistent with LFP measurements of the striatum, thalamus, and STN, and thus has been suggested as a method of distinguishing each structure during electrode placement (Kolb et al., 2017).

### 3.2. DBS of Parkinsonian States

#### 3.2.1. Steady States

**Figure 4:**
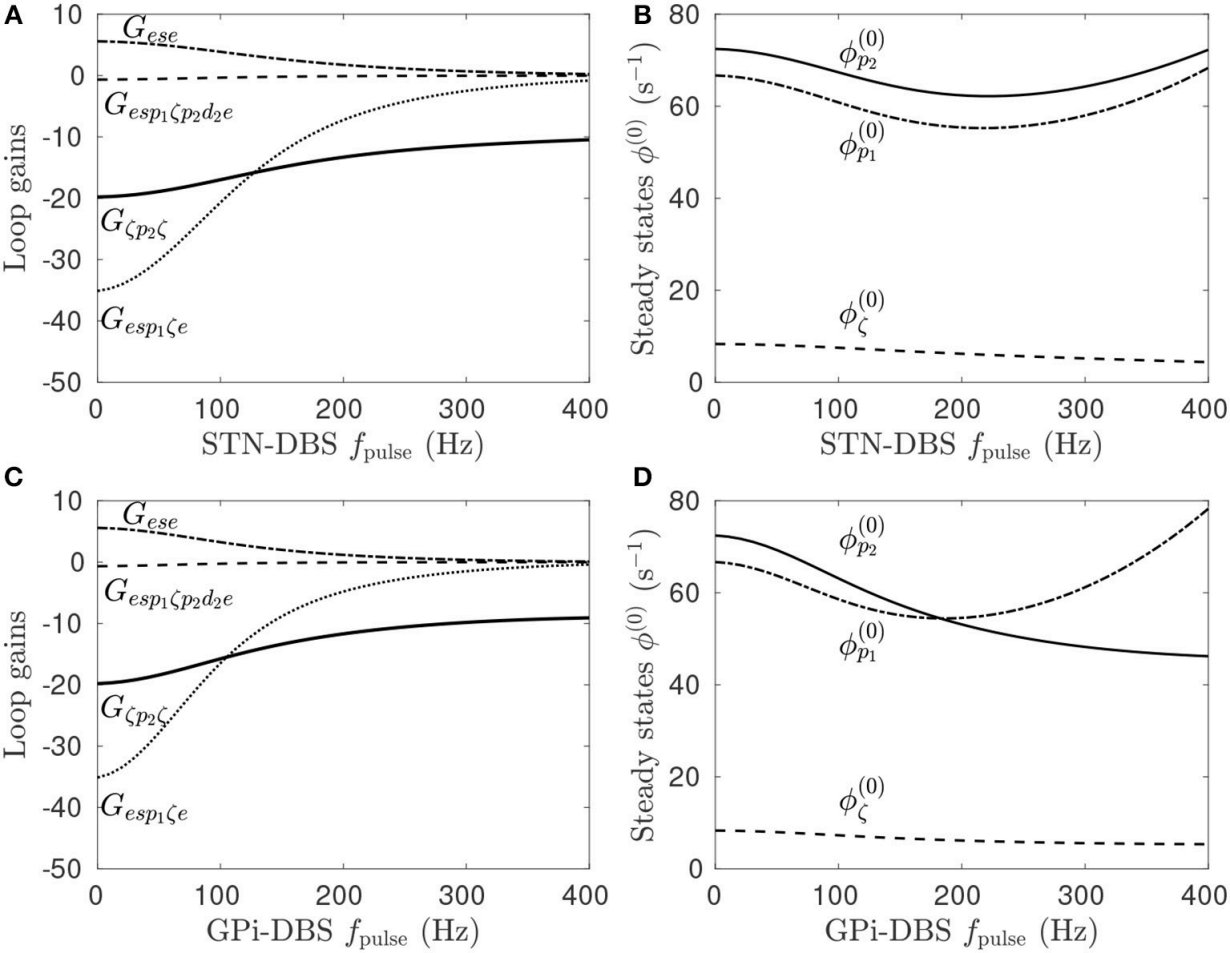
Effects of pulse frequency during STN and GPi stimulation on steady-state firing rates and loop gains. The parameters used are given in Table 1. **(A)** Loop gain dependence on STN-DBS pulse frequency. **(B)** Dependence of steady-state subthalamic and pallidal population firing rates on STN-DBS pulse frequency. **(C)** Loop gain dependence on GPi-DBS pulse frequency. **(D)** Dependence of steady-state subthalamic and pallidal population firing rates on GPi-DBS pulse frequency.

Previous work showed that DBS induced a periodic perturbation of the membrane potential of the stimulus target population (Müller and Robinson, 2018), locked to the stimulus frequency. By averaging over a time window, the perturbation can be approximately represented as a constant value, Δ*V* (Müller and Robinson, 2018), which can then be used to determine the impact of stimulation on the steady-state circuit gains within the network. Pathologically strong gains within the STN-GPe and hyperdirect pathways define the parkinsonian state of the CTBG system and result in enhanced beta activity via a strengthen system resonance (Müller and Robinson, 2018). In Figure 4A it can be seen that regular pulse STN-DBS reduces both the GPe-STN-GPe and hyperdirect loop gains as the *f*_pulse_ increases.

Stimulation of a population in the present model has either an excitatory or inhibitory effect depending on the ratio of synaptic types and strengths adjacent to the stimulating electrode. STN stimulation of parkinsonian states elicits a direct depolarizing effect on the STN population, as defined in Table 3. However, this change in STN activity causes network-wide changes in the activity of all other populations, which in turn alter the inputs to the STN. As such, the stimulation may result in a net hyperpolarization of the STN. This effect can be seen in Figure 4B where, despite an explicit excitatory coupling of the external stimulus to the STN, the steady-state mean STN firing rate is reduced at high *f*_pulse_. Furthermore, stimulation frequencies below 200 Hz hyperpolarize the pallidal populations, which have an explicit excitatory stimulus coupling, and higher frequencies depolarize them.

Figure 4C shows the effect of *f*_pulse_ on the system gains for GPi-DBS is qualitatively similar to the STN-DBS results in Figure 4A. Both the GPe-STN-GPe and hyperdirect loop gains decrease as functions of GPi-DBS *f*_pulse_. The steady-state firing rates for the pallidal populations decrease for *f*_pulse_ < 200 Hz. This result agrees with a study of MPTP-treated monkeys rendered parkinsonian, which showed that 130 Hz GPi stimulation significantly reduced GPi firing rates (Boraud et al., 1996). A key point of difference for GPi-DBS relative to STN-DBS is that the steady-state GPe firing rate continues to decrease for *f*_pulse_>200 Hz.

#### 3.2.2. Temporal Dynamics

**Figure 5:**
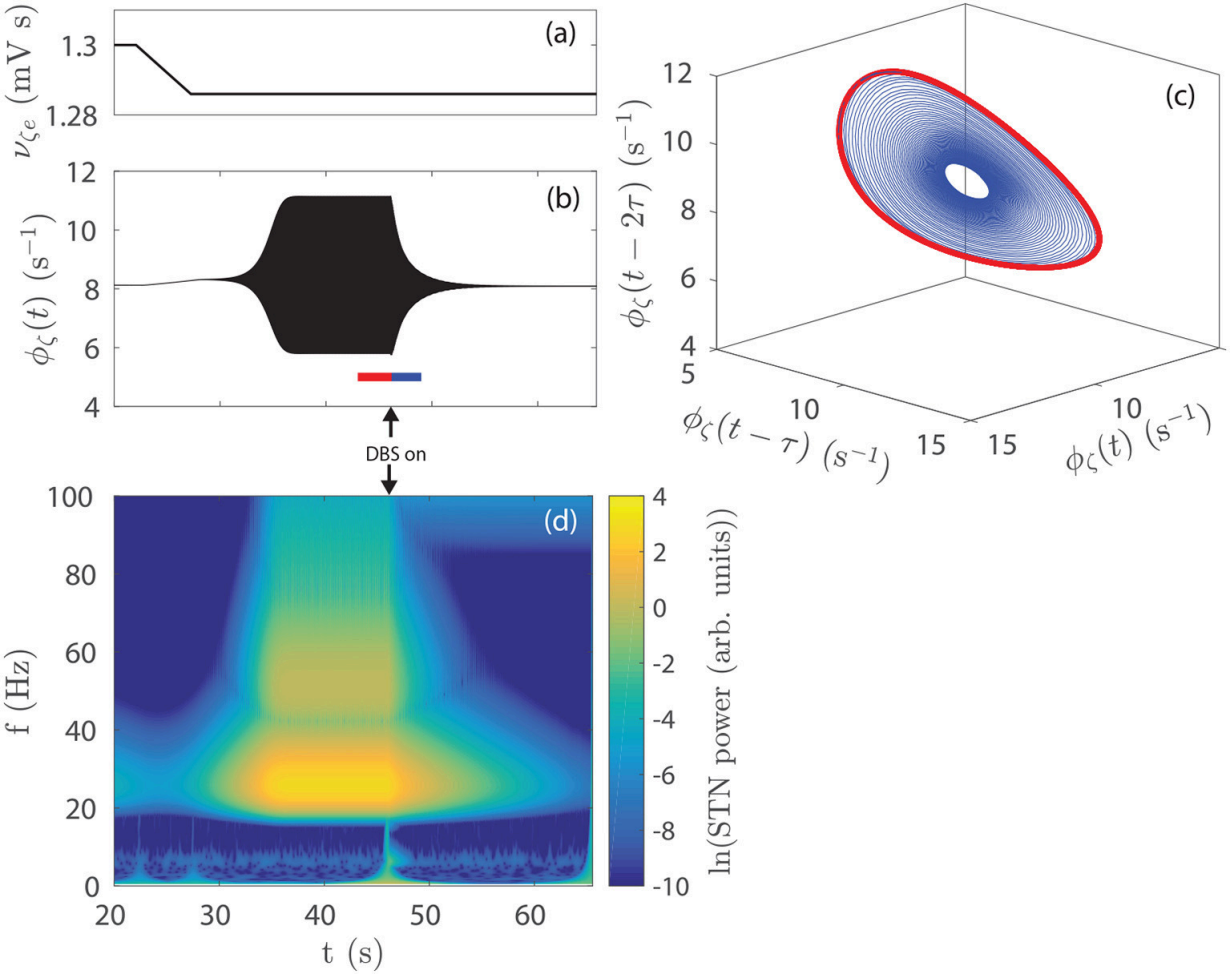
Effect of STN-DBS on parkinsonian beta activity. **(A)** ν_ζ*e*_ is linearly decreased from 1.300 to 1.286 mV s over the time window *t* = 22−27 s. STN-DBS is applied at *t* = 45 s using the 128 Hz pulse function. **(B)** STN firing rate as the system is driven to a regime of dominant beta activity and then suppressed by STN-DBS. The 26 Hz oscillations appear as a solid region because the time scale used renders individual cycles indistinguishable. **(C)** Time-delayed phase portrait of ϕ_ζ_ before stimulation *t* = 43−46 s (red) and during stimulation *t* = 46−49 s (blue). **(D)** STN spectrum as the system is driven to a regime of dominant beta activity which is then suppressed by STN-DBS. Spectrums are calculated using a complex Morlet wavelet transform.

The time series in Figure 5B shows STN activity as subthalamo-cortical coupling is linearly decreased from 1, 300 to 1, 286 mV s, as shown in Figure 5A. The system enters a regime of weakly damped beta resonance where 26 Hz activity dominates. STN-DBS is then applied and a strong damping of the 26 Hz oscillation is observed. Figure 5D shows a spectrogram of this STN time series using a complex wavelet transform with a Morlet wavelet. The amplitude of the STN beta oscillation is reduced by ~80% over the first few seconds following stimulation. After ~10 s, STN activity settles to a mean firing rate close to the value at *t* = 20 s, before ν_ζ*e*_ was decreased. Figure 5C shows a 3-dimensional time-delayed phase portrait of STN activity with no stimulation and during stimulation. The pre-stimulus activity forms a limit-cycle, given by the red line, with an orbital frequency of 26 Hz. The radius of the limit-cycle is proportional to the amplitude of the oscillation in STN firing rate. During stimulation, STN activity still remains on this manifold, however, the amplitude of the orbit is greatly reduced. Although not shown here, when the stimulus is removed the system returns to the limit-cycle attractor with larger amplitude.

#### 3.2.3. Frequency Dependence

**Figure 6:**
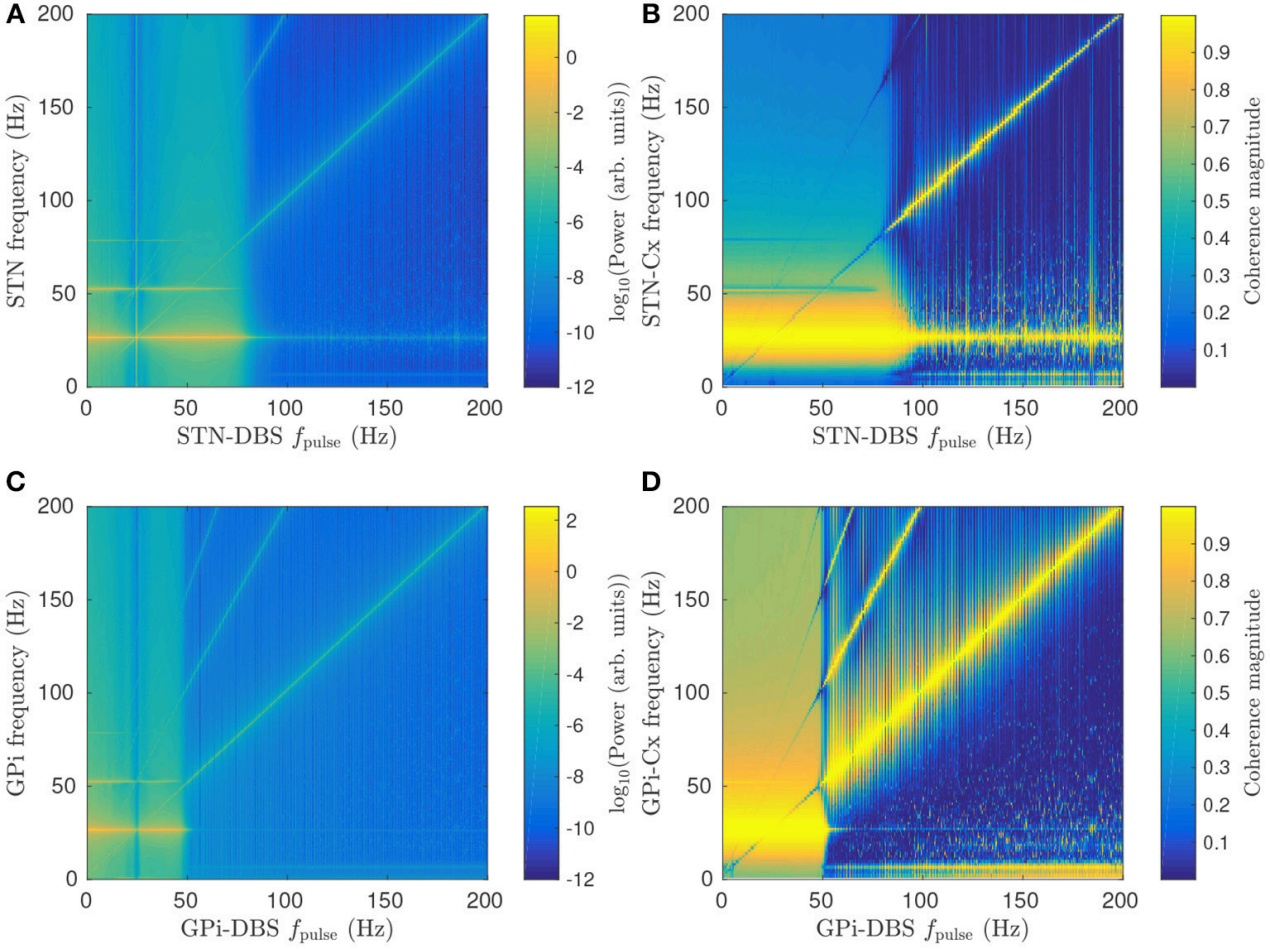
Effects of pulse frequency on parkinsonian beta activity. **(A)** Dependence of STN spectrum on STN-DBS pulse frequency *f*_pulse_. **(B)** Dependence of cortico-STN coherence on STN-DBS pulse frequency *f*_pulse_. **(C)** Dependence of GPi spectrum on GPi-DBS pulse frequency *f*_pulse_. **(D)** Dependence of cortico-GPi coherence on GPi-DBS pulse frequency *f*_pulse_.

Figures 6A,B show the effect of increasing STN-DBS pulse frequency on the STN spectrum and cortico-STN coherence, respectively. Peak power near the beta resonance frequency is seen to drop off sharply for *f*_pulse_>80 Hz. This threshold frequency could be compared to effective frequencies in clinical DBS of PD patients and, in conjunction with EEG and LFP spectrums, used to constrain model parameters in a fitting algorithm such as in the Bayesian approach developed by Abeysuriya and Robinson (2016) for fitting a corticothalamic model to EEG data. Several studies have observed a reduction in cortico-STN coherence during STN-DBS treatments (Kühn et al., 2008; Oswal et al., 2016) and Figure 6B shows this result is consistent with the model.

Figure 6C shows that GPi-DBS has a similar impact on GPi beta activity with a sharp drop in beta power for *f*_pulse_>50 Hz. This threshold pulse frequency is notably smaller than for the STN-DBS case. The difference is due to GPi-DBS having a greater suppressive effect on *G*_*es*_*p*__1_ζ*e*_ than STN-DBS for the same pulse frequency, as shown in Figures 4A,C. Another key difference between GPi-DBS and STN-DBS is that the former has high cortico-GPi coherence for frequencies less than the beta resonance when *f*_pulse_>50 Hz, as shown in Figure 6D, whereas Figure 6C shows a smaller increase in cortico-STN coherence during STN-DBS for the same *f*_pulse_.

During both GPi-DBS and STN-DBS where *f*_pulse_>100 Hz, enhanced < 10 Hz activity is seen in the GPi and STN. This is consistent with enhanced low frequency oscillations in STN LFP recordings following STN-DBS (Priori et al., 2006).

A peak in STN/GPi power and cortico-STN/GPi coherence is also seen in Figures 6A–D at the stimulus pulse frequency and its harmonics. Here, stimulation is driving target population activity, leading to enhanced synchronization at the stimulus frequency. The result was explored in more detail for STN-DBS in a previous study (Müller and Robinson, 2018) and showed good agreement with human EEG studies of steady-state evoked response potentials in the visual cortex during stimulation (Herrmann, 2001).

#### 3.2.4. Random Pulse Effects

**Figure 7:**
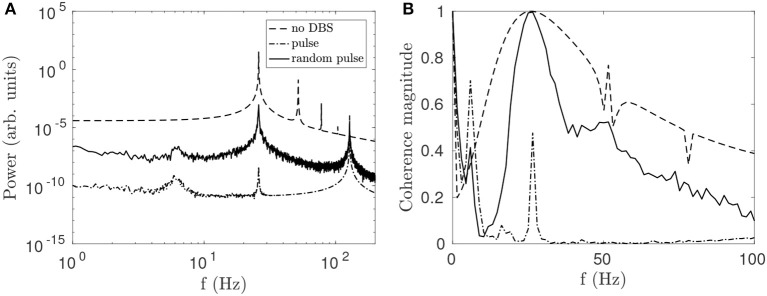
Comparison of effects on STN activity of a regular 128 Hz pulse function and a function of normally distributed pulses with mean frequency *f*_pulse_ = 128 Hz and a standard deviation σ = 4 Hz. **(A)** Effects of pulse and random pulse stimulation on the STN spectrum. The 3 lines plotted are for no DBS (dash), pulse DBS (dash dot), and random pulse DBS (solid), as shown in the legend. **(B)** Effects of pulse and random pulse stimulation on cortico-STN coherence. The 3 lines plotted are defined as in **(A)**.

Randomness is introduced into the pulse function by drawing a new pulse interval from a normal distribution at the end of each pulse. It can be seen in Figure 7A that this variability reduces the suppressive effect of the stimulation on the beta peak relative to the regular pulse function while broadening the entrainment peak. The reduction in beta-band coherence shown in Figure 7B is also less pronounced for the random function. The greater effectiveness of the continuous STN-DBS pulse function relative to the random pulse function for suppressing beta activity is consistent with results from LFP measurements during STN-DBS in a clinical study of human PD (Little et al., 2013).

### 3.3. Response Dependence on Prestimulus State

The parkinsonian state of the CTBG system is defined by large loop gains *G*_*es*_*p*__1_ζ*e*_ and *G*_ζ_*p*__2_ζ_, which result in enhanced beta band activity. In section 3.2.1 it is shown that DBS suppresses this beta activity by reducing the pathologically loop gains when using model parameters defined in Table 1. Here the dependence of stimulus response on the prestimulus state of the model is explored.

For the parameters given in Table 1, increasing ν_*p*_2_ζ_ from 0.6−2.4 mV s increases both *G*_*es*_*p*__1_ζ*e*_ and *G*_ζ_*p*__2_ζ_, and results in large amplitude beta band oscillations. For each value of ν_*p*_2_ζ_ the loop gains and steady state population firing rates are determined for the prestimulus state as well as during 128 Hz pulse DBS, which is done by time-averaging the stimulus induced perturbation to the target membrane potential, as discussed in section 3.2.1 and Müller and Robinson (2018).

**Figure 8:**
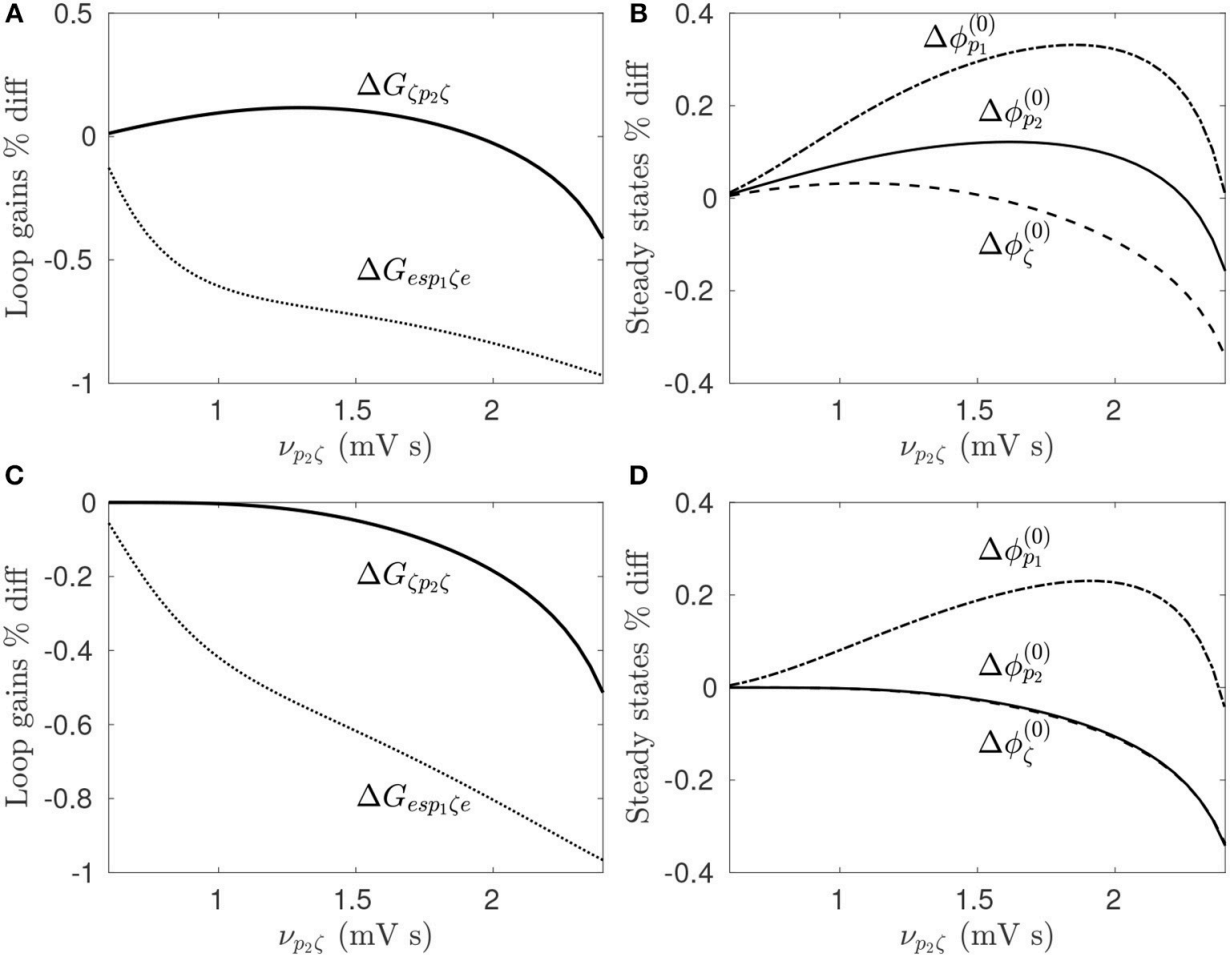
Dependence of stimulus response on prestimulus parameters. For a given value of ν_*p*_2_ζ_ the prestimulus state is compared to the perturbed state during 128 Hz pulse stimulation. **(A)** Percentage difference of prestimulus loop gains relative to perturbed values during STN-DBS. **(B)** Percentage difference of prestimulus steady state firing rates relative to perturbed values during STN-DBS. **(C)** Percentage difference of prestimulus loop gains relative to perturbed values during GPi-DBS. **(D)** Percentage difference of prestimulus steady state firing rates relative to perturbed values during GPi-DBS.

The percentage difference of the loop gains and steady state firing rates between prestimulus states and the perturbed state are shown in Figures 8A,B during STN-DBS and Figures 8C,D during GPi-DBS. Since the stimulation parameters are not being varied here, these figures demonstrate the model predicts neural population response is dependent on prestimulus states of the CTBG system. Figure 8B shows this clearly whereby 128 Hz pulse STN-DBS increases both STN and GPe firing rates for ν_*p*_2_ζ_ < 1.3 mV s and decreases them for ν_*p*_2_ζ_>2.2 mV s. It can also be seen in Figure 8A that the STN-GPe-STN loop gain is increased by 128 Hz pulse STN-DBS for ν_*p*_2_ζ_ < 1.9 mV s and is decreased for values ν_*p*_2_ζ_>1.9 mV s. This result highlights the importance of quantifying prestimulus states of the CTBG system when developing effective DBS treatments, as the same protocols may not have the same impact across patients.

### 3.4. Regional Comparison

The high and low frequency pulse and burst functions, defined in Figure 2 and Table 2, are now used to stimulate the STN and GPi. The impact of each of these stimulation protocols on parkinsonian beta activity and coherence measures is then compared.

#### 3.4.1. STN Activity and Coherence

**Figure 9:**
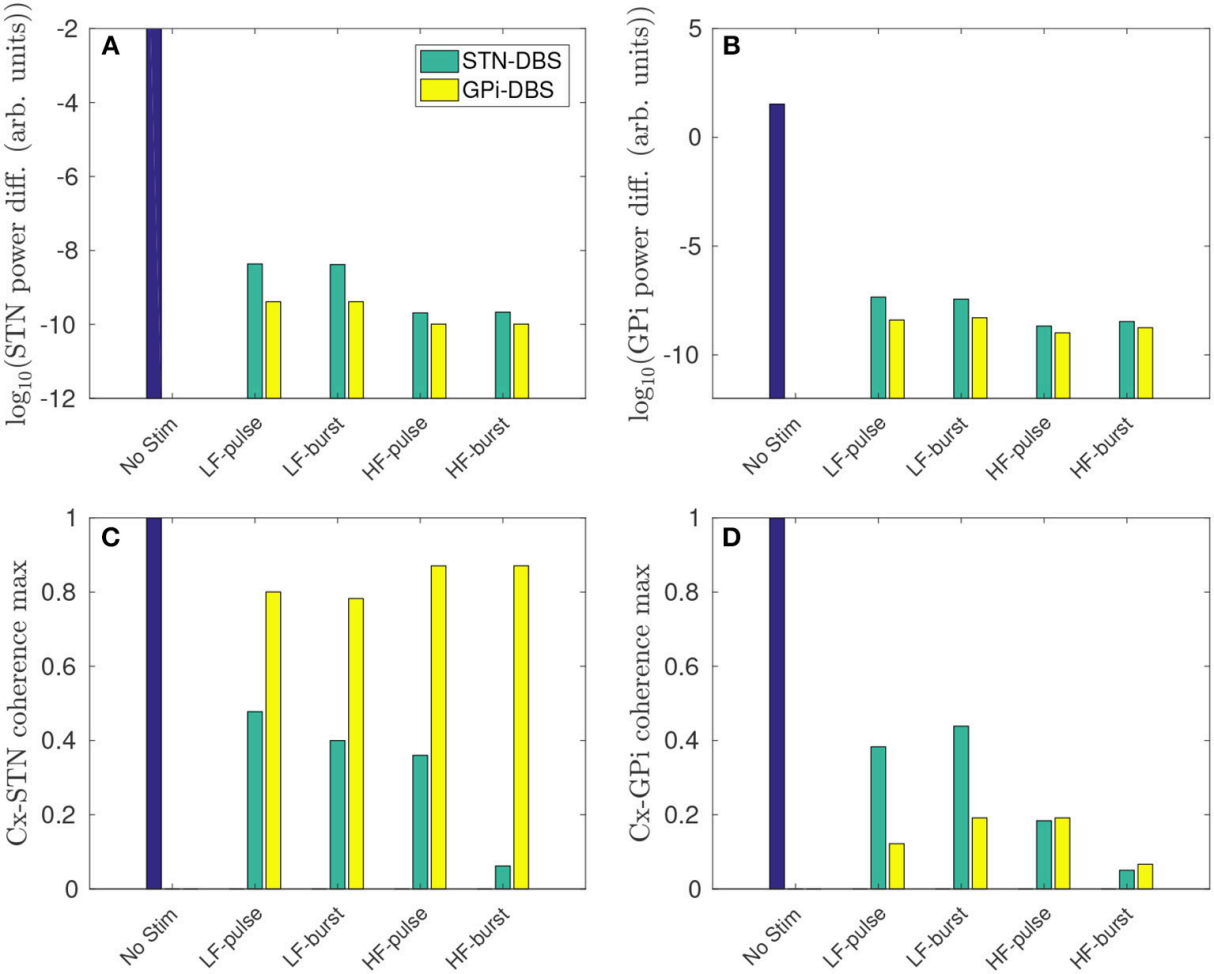
Regional comparison of stimulus protocols on parkinsonian beta activity in the STN and GPi. The stimulus protocols are defined in Figure 2 and Table 2. The bars depict the case for no stimulus (blue), and during STN-DBS (green) and GPi-DBS (yellow). **(A)** Effect of stimulus protocols on the power difference (maximum - minimum) of STN activity in the beta band. **(B)** Effect of stimulus protocols on the power difference (maximum - minimum) of GPi activity in the beta band. **(C)** Effect of stimulus protocols on cortico-STN magnitude coherence within the beta band. **(D)** Effect of stimulus protocols on cortico-GPi magnitude coherence within the beta band.

As shown in Figure 9A, both STN-DBS and GPi-DBS suppress peak STN power within the beta frequency band. GPi-DBS is slightly more effective for all stimulus protocols tested. Despite GPi outputs not being directly connected to the STN, GPi-DBS is able to induce significant changes in STN firing patterns, specifically in the beta band, while Figure 4D shows only a correspondingly small change is induced in the mean STN population firing rate.

A higher pulse frequency, using either the pulse or burst function, produced a stronger beta suppression. This is expected since a higher number of pulses will drive a larger mean perturbation to the membrane potential of the stimulated populations.

A reduction of cortico-STN coherence within the beta band is also observed for both STN-DBS and GPi-DBS. However, as shown in Figure 9C, GPi-DBS is less effective than STN-DBS.

STN-DBS using the high frequency burst function suppressed peak STN activity and cortico-STN coherence in the beta band most effectively out of the stimulus protocols compared.

Overall, GPi-DBS is more effective than STN-DBS for suppressing beta activity in the STN, but less effective at reducing cortico-STN beta-band coherence. However, it is not clear which of these measures, power of STN activity or cortico-STN coherence, is most strongly correlated with PD motor symptoms.

#### 3.4.2. GPi Activity and Coherence

Figure 9B shows that peak beta activity in the GPi is suppressed during GPi-DBS and STN-DBS, and higher stimulation frequencies yield stronger suppression. The suppression of GPi beta activity during >50 Hz STN-DBS agrees with experimental results of GPi LFP spectrum in human PD patients (Brown et al., 2004; Kühn et al., 2008).

Cortico-GPi coherence is also reduced for all stimulus protocols tested, as shown in Figure 9D. The pulse function for GPi-DBS proved most effective for decreasing beta coherence at low stimulation frequency. As for the STN, the high frequency burst function is most effective for suppressing beta band activity in the GPi and reducing cortico-GPi coherence. The model result is consistent with a study of human PD which showed elevated cortico-pallidal coherence in the beta band, and that this coherence was reduced during therapeutic GPi-DBS (Wang et al., 2018).

### 3.5. Combined STN and GPi DBS

Several experimental studies have suggested that simultaneous stimulation of multiple nuclei may be more therapeutically effective than stimulating a single nucleus (Mazzone et al., 2005; Oertel et al., 2017). To test this in the present model, the connection structures for the individual cases of STN-DBS and GPi-DBS, described in section 2.10, are combined. For comparison, the pulse amplitude of the dual STN/GPi-DBS is defined to be half that of their respective individual forms; i.e., the two nuclei each receive half the amplitude.

**Figure 10:**
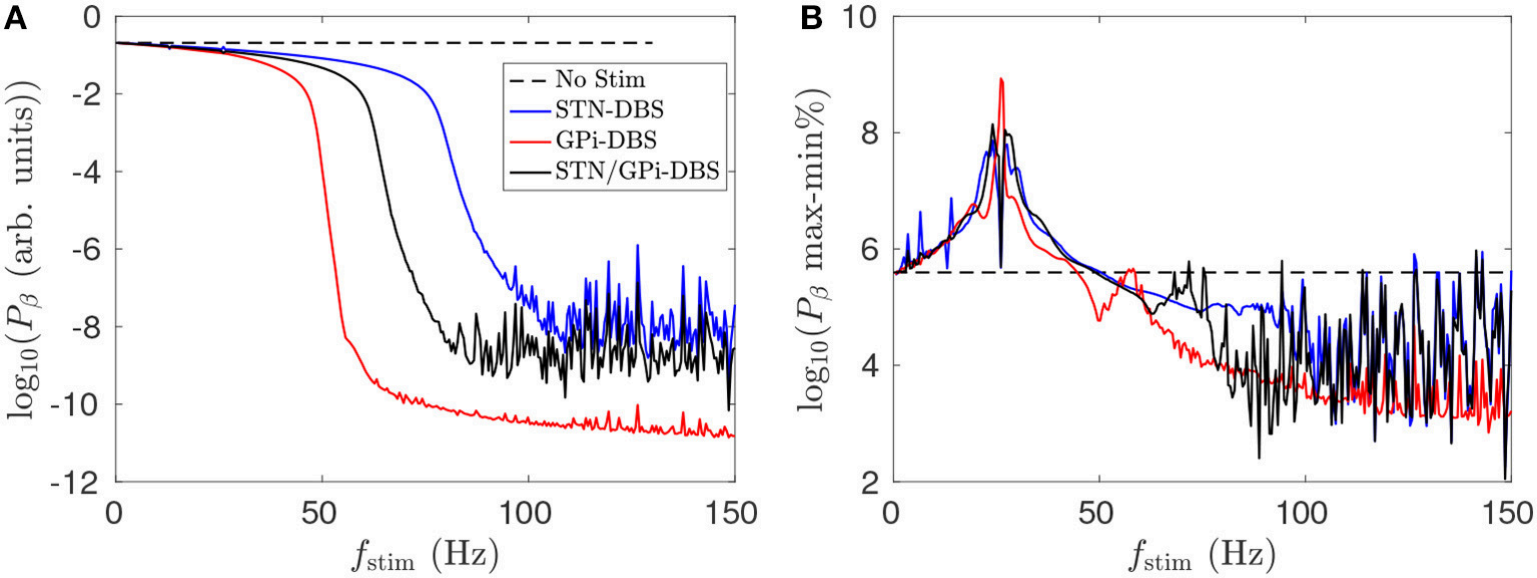
Comparison of dual STN/GPi-DBS, STN-DBS, and GPi-DBS for suppressing STN beta activity *P*_β_. **(A)** Frequency dependence of mean STN beta band activity during STN/GPi-DBS (black), STN-DBS (blue), and GPi-DBS (red) using pulse stimulation. **(B)** Frequency dependence of percentage difference between maximum and minimum STN beta band activity during STN/GPi-DBS (black), STN-DBS (blue), and GPi-DBS (red) using pulse stimulation.

Figure 10A shows that dual STN/GPi-DBS is less effective than GPi-DBS for reducing mean STN beta activity. If the pulse amplitude of dual STN/GPi-DBS is instead not reduced and has the same amplitude as the individual forms, the dual STN/GPi-DBS frequency dependence shown by a black line in Figure 10A shifts to the left and becomes more effective than GPi-DBS at low frequencies. However, dual STN/GPi-DBS still approaches the STN-DBS case, shown by the blue line in Figure 10A, as *f*_stim_ increases.

In application, dual STN/GPi-DBS would allow simultaneous LFP to be recorded from the STN and GPi via the stimulating electrodes. This could permit a better measure of the efficacy of stimulation that incorporates some combination of induced change in activity of the two populations not considered here.

Figure 10B shows that for *f*_stim_ < 50 Hz the percentage difference between the maximum and minimum STN beta band power is higher than when no stimulation is applied. Clinical studies have observed worsening motor symptoms during STN-DBS stimulation at frequencies < 30 Hz (Fogelson et al., 2005a; Chen et al., 2007; Eusebio et al., 2008). Our results suggest this clinical observation could be due to a sharpened beta peak caused by the low frequency stimulation. Even though the power of STN beta activity is slightly reduced during *f*_stim_ < 50 Hz stimulation, excluding the constructive wave interactions at the resonance frequency and subharmonics described in Müller and Robinson (2018), the power is more focused at particular beta frequencies. A measure of this sort could be explored as an LFP marker for adaptive stimulation treatments.

Although not shown here, dual STN/GPi-DBS with *f*_stim_>100 Hz was more effective at reducing maximum cortico-STN beta band coherence than the respective individual forms at *f*_stim_>100 Hz. Additionally, introducing a phase shift between the STN-DBS and GPi-DBS components, which comprise dual STN/GPi-DBS, produces no discernible difference in population activity. This is likely due to the much shorter stimulation time scales than those of each population response.

The overall suppressive effect of dual STN/GPi-DBS is a combination of the individual STN-DBS and GPi-DBS results shown in Figure 4. Stimulation induces a mean perturbation to the membrane potentials of the target populations, the STN and GPi, as well as the populations they project to. This results in a reduction of the *G*_*es*_*p*__1_ζ*e*_ and *G*_ζ_*p*__2_ζ_ loop gains which are responsible for the beta resonance.

### 3.6. Effects of Pulse Parameters

**Figure 11:**
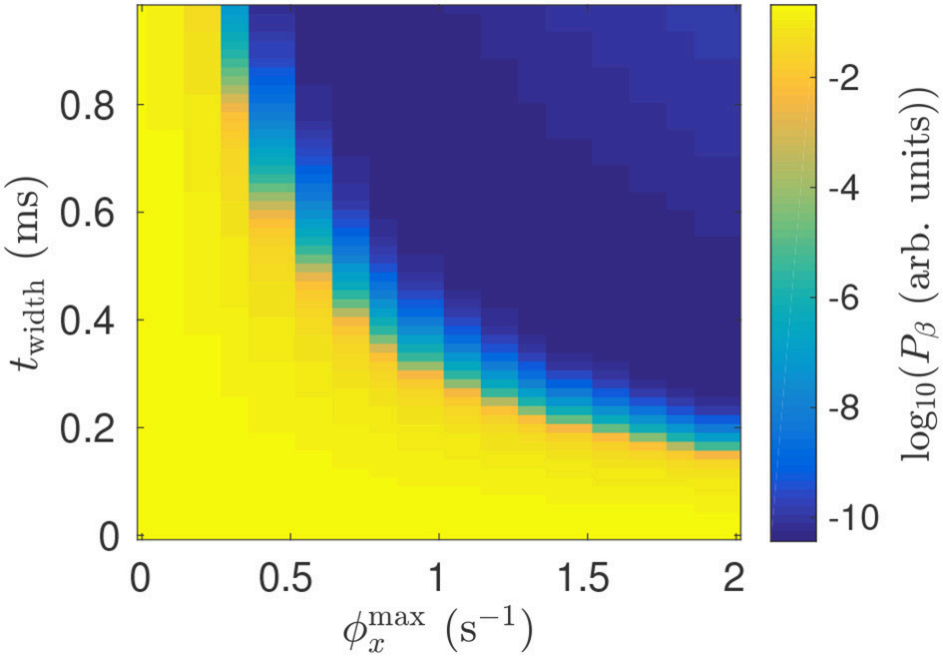
Dependence of mean STN beta band power *P*_β_ during 128 Hz STN-DBS on the pulse width and pulse amplitude.

The model shows that the suppressive effect of DBS on beta activity is due to a mean perturbation of population membrane potentials. This perturbation is dependent on the stimulus pulse frequency, as well as the pulse amplitude, and pulse width. Figure 11 shows impact of stimulation pulse amplitude and width on the mean STN beta band activity. This activity is decreased by several orders of magnitude for $t_{\text{width}}\phi_{x}^{\text{max}}\leq 5\times 10^{-4}$.

### 3.7. Theta Oscillations

Parkinsonian beta oscillations in the present model result from pathologically large loop gains in the STN-GPe and hyperdirect pathways. Decreasing the STN-GPe and cortico-STN coupling strengths from the parkinsonian estimates in Table 1 toward nominal estimates from a previous study (van Albada and Robinson, 2009) leads to a decrease in the GPe-STN loop gain, but a smaller decrease in the hyperdirect loop gain. This results in a dominant resonance which drives 6 Hz activity in the system. Studies have found STN activity and cortico-STN coherence in the beta band are both reduced during tremor (Qasim et al., 2016) and thus suggest that parkinsonian tremor may be a side effect of a similar naturally occurring mechanism to reduce pathologically large beta oscillations.

**Figure 12:**
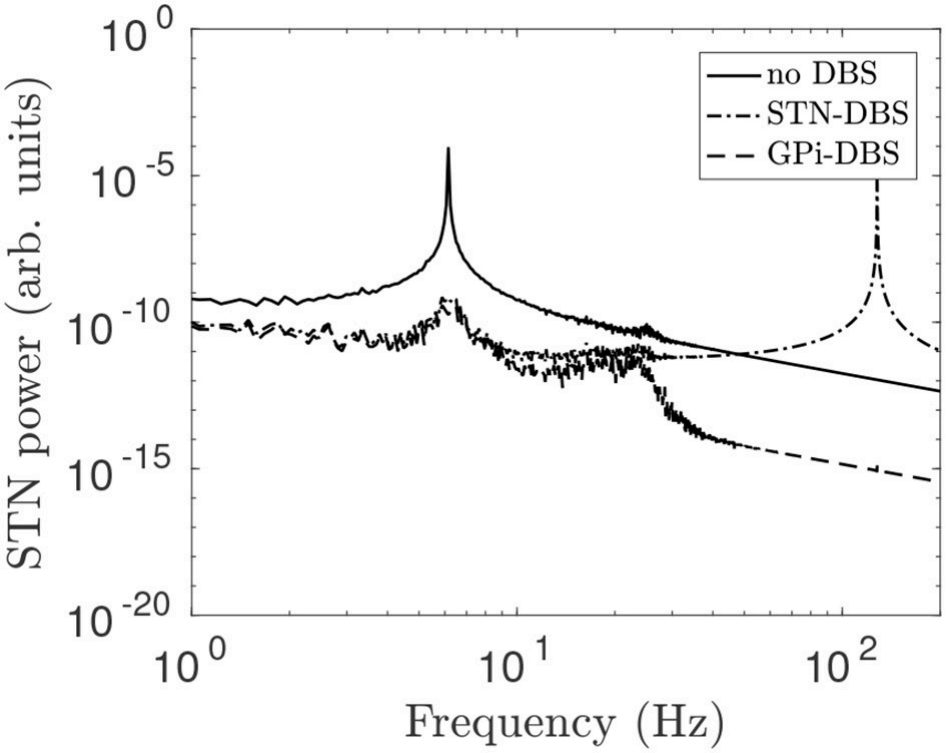
STN power spectrum during STN-DBS and GPi-DBS using regular 128 Hz pulse stimulation. The system is configured to generate theta activity by changing the coupling parameters ν_ζ*e*_ = 0.915 mV s and ν_*p*_2_ζ_ = 2 mV s and using all other parameters from Table 1. The line types distinguish STN power spectrums for no stimulation (solid), during STN-DBS (dash dot), and during GPi-DBS (dash).

In Figure 12, STN-GPe and cortico-STN coupling strengths are reduced and this drives enhanced 6 Hz theta band oscillations in STN activity. STN-DBS and GPi-DBS, using a 128 Hz pulse, is then applied and suppresses the 6 Hz STN spectral peak. This is consistent with clinical trails of STN-DBS in essential tremor patients who showed marked tremor reduction during stimulation (Blomstedt et al., 2010, 2011).

## 4. Discussion

We have developed a neural field theory of deep brain stimulation of the subthalamic nucleus and the globus pallidus internus and applied it to parkinsonian states of the corticothalamic-basal ganglia system. The theory allows systematic determination of effective stimulus protocols for suppressing pathological rhythms and synchronization in Parkinson's disease. The key results of the work are as follows:

(i) The impact of stimulation is found to be dependent on prestimulus states of the CTBG system. DBS is approximated as an activation of afferent and efferent axons in close proximity to the stimulating electrode which results in a net perturbation to mean population membrane potentials. This perturbation may be excitatory (depolarizing) or inhibitory (hyperpolarizing) depending on the ratio of synaptic types adjacent to the stimulating electrode. An explicit excitatory or inhibitory coupling of an external stimulus to a target population induces network-wide changes in activity, affecting inputs to the stimulus target, and may result in a net perturbation to target population activity that is opposite to the explicit stimulus coupling. A change in brain state, such as between rest and movement, may mean the system has shifted from a region where DBS protocols are therapeutically efficacious to one where they no longer are. Adaptive DBS systems could use a model such as the present one to track brain states and determine what stimulation protocols are most effective for that state. LFP power in the beta band is already being explored as a marker for when to apply stimulation (Little et al., 2013). However, by fitting a model to patient data, a region of state space could be defined on the basis of multiple biomarkers, such as particular combinations of state variables, but importantly the trajectory of these state variables as well. This could allow for earlier detection of motor symptom onset and adaptive DBS to provide therapeutic benefit with lower stimulation power.

(ii) Pathological beta activity in the STN and GPi was suppressed during both STN-DBS and GPi-DBS. A stimulation evoked perturbation to the mean membrane potentials of the target population and its projection sites reduced pathologically large loop gains in the hyperdirect and STN-GPe pathways. Beta activity in the GPi was reduced during STN-DBS, which is consistent with experimental findings (Brown et al., 2004; Kühn et al., 2008). The threshold stimulus pulse frequency required for effective damping of the beta oscillations was about 80 Hz for STN-DBS and 50 Hz for GPi-DBS. This threshold frequency could be compared to effective frequencies in clinical DBS of PD patients and, in conjunction with EEG and LFP spectrums, used to constrain model parameters in a fitting algorithm such as in Abeysuriya and Robinson (2016). Entrainment of target population activity to the stimulus frequency and its harmonics was also observed, which have been explored in more detail in a previous study (Müller and Robinson, 2018).

(iii) A reduction of cortico-STN coherence resulted during STN-DBS which agrees with findings of human PD studies (Kühn et al., 2008; Oswal et al., 2016). Another study showed cortico-GPi coherence was reduced during GPi-DBS in human PD (Wang et al., 2018) and this is consistent with the model results.

(iv) The burst function during STN-DBS and GPi-DBS was consistently more effective than the regular pulse function with the same number of pulses for suppressing beta activity in the STN and GPi, as well as reducing cortico-STN coherence. Randomness was introduced to the pulse function using a mean pulse frequency equal to the pulse frequency of the regular function. The random function proved less effective than the regular one, in accord with human PD experiments (Little et al., 2013). The random function also produced a broader entrainment peak and reduced its amplitude.

(v) The GPi was the most effective DBS target for reducing beta activity across the STN and GPi and cortico-GPi beta band coherence. However, DBS targeting the STN was most effective for reducing cortico-STN coherence in the beta band. Clinical studies of STN-DBS and GPi-DBS have shown similar long term outcomes on the motor symptoms in PD (Moro et al., 2010); however, STN-DBS allows for greater medication reduction (Liu et al., 2014) and GPi-DBS has shown fewer adverse effects (Moro et al., 2010). Coherence measures between the motor cortex and basal ganglia may prove to be useful biomarkers for PD, and for present and future adaptive DBS systems used in symptom treatment. Exploring the impact of different stimulus protocols on these coherence measures could lead to improved treatment efficacy.

(vi) Low frequency STN-DBS (< 50 Hz) was found to result in less distributed STN activity within the beta frequency band. Clinical studies have observed worsening motor symptoms during STN-DBS stimulation at frequencies < 30 Hz (Fogelson et al., 2005a; Chen et al., 2007; Eusebio et al., 2008). Our results suggest this clinical observation could be due to a sharped beta peak, with STN activity more focused at particular beta band frequencies during low frequency stimulation.

(vii) Dual stimulation of the STN and GPi proved less effective than GPi-DBS at reducing STN beta activity when conserving pulse amplitude, however, it was more effective at reducing cortico-STN beta band coherence.

(viii) A reduction of the pathologically large GPe-STN and cortico-STN coupling strengths, which define parkinsonian states of the CTBG system, resulted in dominant 6 Hz activity. Studies have shown that, during tremor, beta band STN activity and cortico-STN coherence are reduced (Qasim et al., 2016), which suggests that parkinsonian tremor may be a side effect of a compensatory mechanism for reducing pathologically large beta oscillations. Both STN-DBS and GPi-DBS using a 128 Hz pulse frequency suppressed theta band STN activity in the model. Stimulation of the ventral intermediate nucleus of the thalamus and STN have proved effective for reducing parkinsonian tremor in human PD (Krack et al., 1998; Hariz et al., 2008). However, STN LFP recordings covering the 4−6 Hz tremor frequency have yet to be demonstrated as useful for tremor detection (Hirschmann et al., 2017).

Overall, the present study reproduces and unifies existing experimental and clinical results on large scale measures of brain activity such as EEG and LFP, and allows systematic comparison of the effectiveness of deep brain stimulation protocols and targets for suppressing parkinsonian rhythms in the corticothalamic-basal ganglia system. Further work might include fitting the model to EEG and LFP data in order to estimate patient specific model parameters, which would allow for an optimized stimulation protocol to be developed. Abeysuriya and Robinson (2016) developed a Bayesian approach using a Metropolis-Hastings algorithm for model parameter fitting of a corticothalamic system to EEG data and a similar approach could be used for the corticothalamic-basal ganglia model.

## Author Contributions

EM developed the model, performed data analysis, and drafted the manuscript. PR analyzed the data and helped formulate and revise the manuscript.

### Conflict of Interest Statement

The authors declare that the research was conducted in the absence of any commercial or financial relationships that could be construed as a potential conflict of interest.
